# Unraveling the role of HIF-1α in sepsis: from pathophysiology to potential therapeutics—a narrative review

**DOI:** 10.1186/s13054-024-04885-4

**Published:** 2024-03-27

**Authors:** Hang Ruan, Qin Zhang, You-ping Zhang, Shu-sheng Li, Xiao Ran

**Affiliations:** 1 Department of Critical-Care Medicine, Tongji Hospital, Tongji Medical College, Huazhong University of Science and Technology, Jiefang Ave, Wuhan, 430030 People’s Republic of China; 2 Department of Emergency Medicine, Tongji Hospital, Tongji Medical College, Huazhong University of Science and Technology, Wuhan, China; 3 Department of Anesthesiology, Hubei Key Laboratory of Geriatric Anesthesia and Perioperative Brain Health, and Wuhan Clinical Research Center for Geriatric Anesthesia, Tongji Hospital, Tongji Medical College, Huazhong University of Science and Technology, Wuhan, 430030 China

**Keywords:** Sepsis, Critical care, HIF-1α, Hypoxia, Molecular medicine

## Abstract

Sepsis is characterized by organ dysfunction resulting from a dysregulated inflammatory response triggered by infection, involving multifactorial and intricate molecular mechanisms. Hypoxia-inducible factor-1α (HIF-1α), a notable transcription factor, assumes a pivotal role in the onset and progression of sepsis. This review aims to furnish a comprehensive overview of HIF-1α's mechanism of action in sepsis, scrutinizing its involvement in inflammatory regulation, hypoxia adaptation, immune response, and organ dysfunction. The review encompasses an analysis of the structural features, regulatory activation, and downstream signaling pathways of HIF-1α, alongside its mechanism of action in the pathophysiological processes of sepsis. Furthermore, it will delve into the roles of HIF-1α in modulating the inflammatory response, including its association with inflammatory mediators, immune cell activation, and vasodilation. Additionally, attention will be directed toward the regulatory function of HIF-1α in hypoxic environments and its linkage with intracellular signaling, oxidative stress, and mitochondrial damage. Finally, the potential therapeutic value of HIF-1α as a targeted therapy and its significance in the clinical management of sepsis will be discussed, aiming to serve as a significant reference for an in-depth understanding of sepsis pathogenesis and potential therapeutic targets, as well as to establish a theoretical foundation for clinical applications.

**Graphical Abstract:**

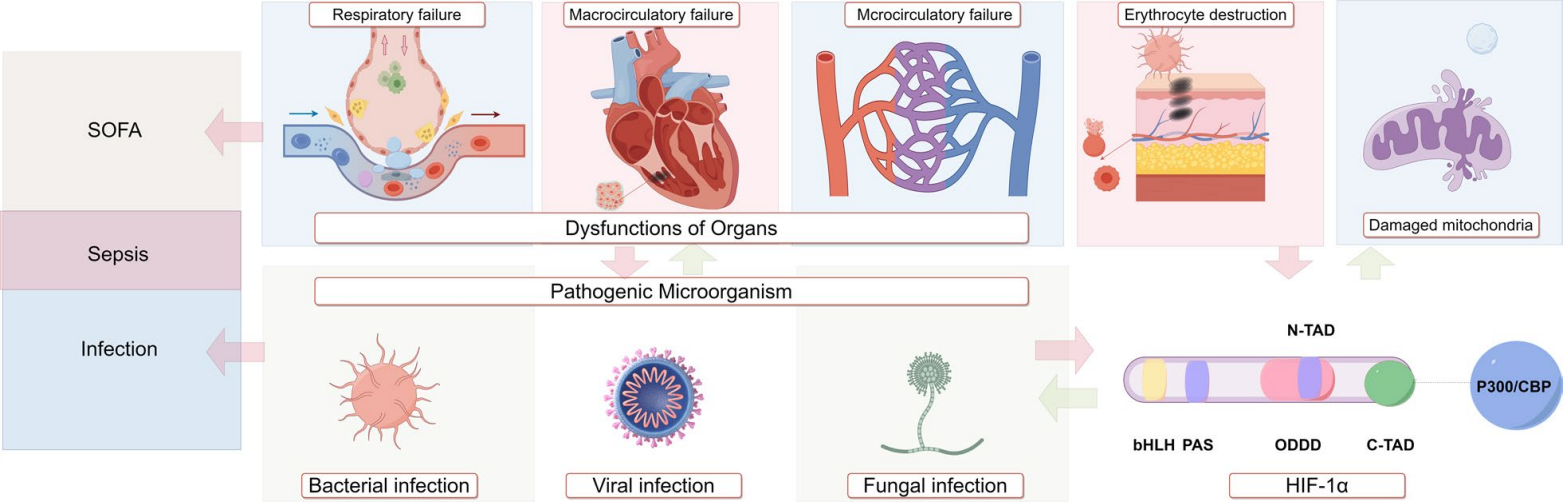

## Background

Sepsis is a complex and multifaceted condition triggered by infection, leading to a series of pathological, physiological, and molecular alterations in the body [1]. As both basic and clinical research on sepsis advance, the understanding and characterization of this condition undergo continuous refinement and expansion. The evolution of the sepsis definition can be traced from its initial description in Sepsis 1.0, which defined sepsis as systemic inflammatory response syndrome (SIRS) resulting from infection [2]. This progressed in Sepsis 2.0, which integrated clinical symptoms and signs into the assessment of Sepsis 1.0[3]. Subsequently, Sepsis 3.0 redefined sepsis as a life-threatening condition arising from an uncontrolled inflammatory response to infection, coupled with life-threatening organ dysfunction caused by the same [4]. The evolution of the concept of sepsis mirrors an advancement in the understanding of the disease, shifting from the examination of external signs and symptoms to the exploration of molecular-level abnormalities inherent to sepsis.

Hypoxia is a prevalent pathophysiological alteration observed in sepsis. Heightened inflammatory responses increase vascular permeability, resulting in acute pulmonary edema and subsequent development of Acute Respiratory Distress Syndrome (ARDS) [5]. Moreover, organ dysfunction in septic patients arises from ineffective perfusion to organ tissues due to vascular endothelial damage, cellular dysfunction, and activation of the coagulation system, leading to intravascular microthrombosis and subsequent macrocirculatory and microcirculatory failure [6]. Furthermore, inadequate perfusion exacerbates hypoxia and compromises cellular oxygen utilization [7]. Hypoxia acts as the link connecting the pathophysiological changes in sepsis to the molecular alterations of hypoxia-inducible factor-1α (HIF-1α). Specifically, HIF-1α levels are intricately regulated by oxygen, undergoing degradation under normoxic conditions and accumulation in hypoxic environments [8]. HIF-1α is a heterodimeric protein complex that serves as a critical regulator of the cellular response to physiological hypoxia and infection, exerting diverse pathophysiological effects at the cellular, tissue, and organismal levels [9–11]. The aim of this review is to elucidate the role of HIF-1α and its related mechanisms in the initiation, progression, and immune response of sepsis, as well as to evaluate its potential therapeutic implications.

## Molecular biology of HIF-1α

### Basic concepts of HIF-1α

Hypoxia-inducible factor (HIF) is a heterodimeric protein complex consisting of a constitutively expressed subunit *β* and an oxygen-dependent subunit α [11]. In mammals, the α-subunit is found in three isoforms: HIF-1α, HIF-2α, and HIF-3α [12]. HIF-1α is typically linked to acute hypoxia and is accountable for activating glycolytic genes, reducing oxygen consumption, and alleviating reactive oxygen species (ROS) production [8].

HIF-1α belongs to the basic Helix-Loop-Helix-Periodicity-Aryl Hydrocarbon Receptor Nuclear Translocator-Single-Minded (bHLH-PAS) family, which includes bHLH and PAS protein structural domains [13]. The bHLH-PAS motifs enable HIF-1α and HIF-1β to form a dimer, facilitating their binding of HIF to hypoxia response elements (HRE) on target genes [12, 13]. Additionally, these motifs aid in promoting the binding of HIF to HREs on target genes and consist of two transactivation domains (TAD): the NH2-terminal domains (N-TAD) and the COOH-terminal domains (C-TAD) [13]. The N-TAD stabilizes HIF-1α and prevents degradation [13]. Cyclic adenosine monophosphate-response binding protein binding protein (CBP) and p300 are two closely related histone acetyltransferase (HAT) enzymes capable of binding to the C-TAD, acting as binding proteins to regulate HIF-1α transcription under hypoxic conditions [14].

HIF-2α primarily functions in chronic hypoxia [15]. The stability of *HIF-2α* mRNA levels exceeds that of *HIF-1α* mRNA [16]. HIF-2α enhances erythropoietin (EPO) synthesis, manages iron metabolism, regulates fatty acid synthesis and uptake, and significantly influences chronic inflammation, fibrosis, and tumorigenesis [8]. Although less explored than HIF-1α and HIF-2α, HIF-3α also holds significance in the hypoxia response. HIF-3α possesses a transcriptional regulatory function and competes with HIF-1α and HIF-2α for binding to the transcriptional elements of target genes during hypoxia, thus exerting a negative regulatory influence on the expression of genes associated with the HIF pathway [17].

### Oxygen-dependent regulatory pathway of HIF-1α

Although HIF-1α is widely expressed in cells, it undergoes rapid degradation in vivo under normoxia (21% oxygen) [13, 18, 19]. On HIF-1α, three hydroxylation sites exist: an oxygen-dependent degradation domain (ODDD) that overlaps with the N-TAD and encompasses two proline residues, as well as one asparaginyl residue in the C-TAD [12]. In a cellular environment rich in oxygen, HIF-1α undergoes oxygen-dependent prolyl-4-hydroxylase (PHD)-mediated hydroxylation of proline residues [13]. PHD, also known as prolyl hydroxylase, consists of three isoforms: PHD1, PHD2, and PHD3, serving as crucial enzymes in the cell by identifying and hydroxylating proline residues in proteins for modification. This hydroxylation modification of proline enables its interaction with von Hippel–Lindau protein (pVHL), an E3 ubiquitin ligase capable of selectively degrading HIF-1α [12].

**Fig. 1:**
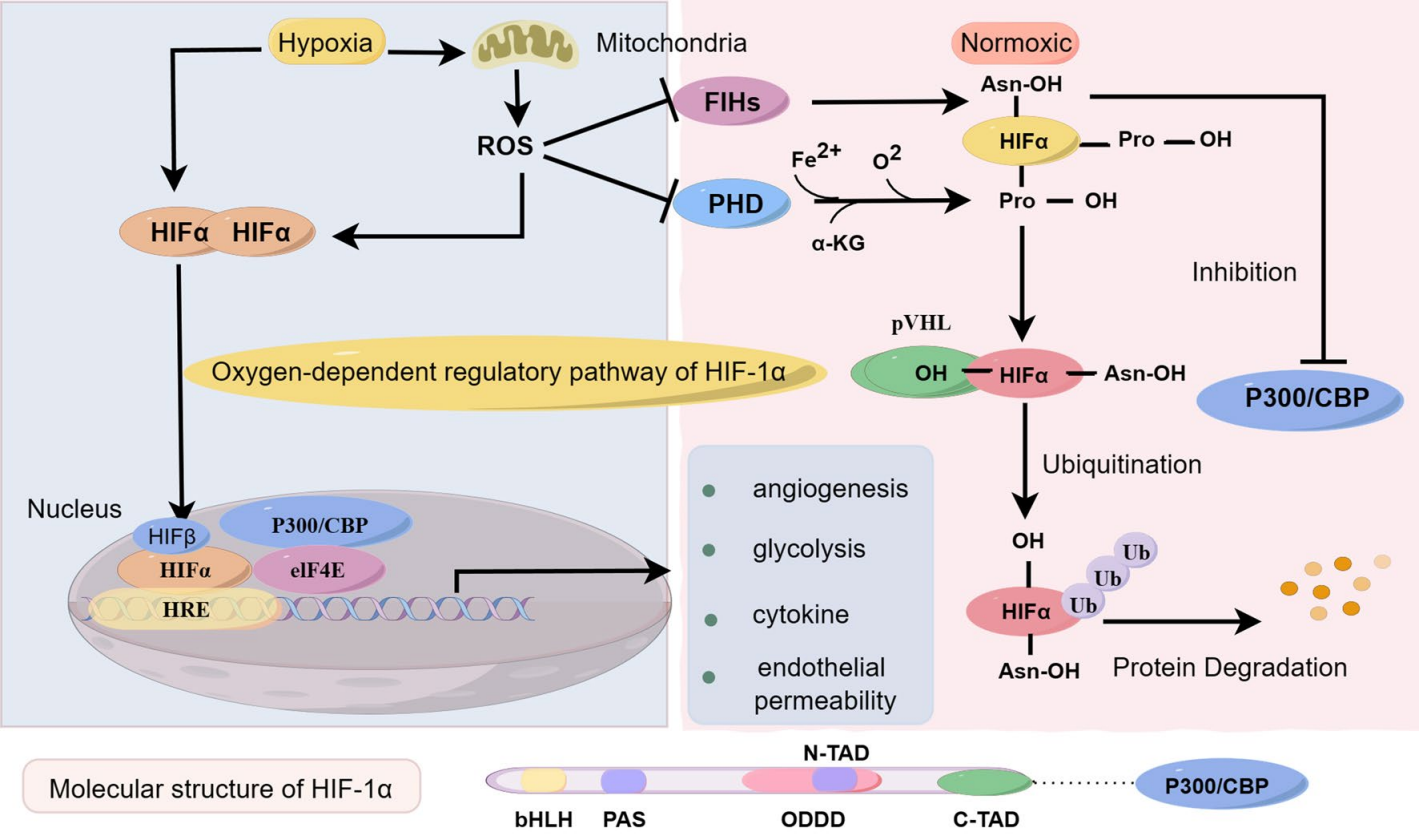
Oxygen-dependent regulatory pathway of HIF-1α. (1) The oxygen-dependent regulatory pathway of HIF-1α encompasses two mechanisms: Under normal oxygen levels, the stability of HIF-1α is controlled by intracellular prolyl hydroxylase (PHD), which modifies specific proline residues on HIF-1α through hydroxylation when oxygen levels are sufficient. The VHL protein (Von Hippel–Lindau protein) is also involved in oxygen-dependent regulation, leading to the ubiquitination and subsequent degradation of HIF-1α. However, under hypoxic conditions or reduced oxygen levels, decreased PHD activity diminishes the degradation of HIF-1α, resulting in increased protein stability and the capacity to enter the nucleus and activate HIF-1α-dependent gene transcription. (2) Factor-inhibiting HIF (FIH) protein is another crucial oxygen-dependent regulatory protein. Under normoxic conditions, FIH restricts the transcriptional activity of HIF-1α by hydroxylating aspartic acid residues on HIF-1α, thereby impeding its binding to the transcriptional cofactor p300/CBP. Conversely, under hypoxic conditions, decreased activity of FIH allows HIF-1α to enhance its transcriptional activity by binding to p300/CBP. EIF4E, HIF-1α, HIF-1β, and the coactivators p300/CBP form a complex to activate downstream target genes as part of the hypoxia response. Under low oxygen conditions, HIF-1α stability is enhanced, enabling it to translocate to the cell nucleus, where it dimerizes with HIF-1β to form the HIF-1 complex. This complex binds to hypoxia response elements in the promoter regions of target genes. Concurrently, p300/CBP coactivators interact with the HIF-1 complex, enhancing its transcriptional activity. EIF4E, a key post-transcriptional regulatory factor, interacts with the HIF-1/p300/CBP complex, modulating the translation of specific mRNAs, and resulting in enhanced protein synthesis. This collective action serves to activate downstream target genes involved in the cellular response to low oxygen levels and related biological processes

Furthermore, under oxygen-sufficient conditions, HIF-α is suppressed by factor-inhibiting HIF-1 (FIH) through FIH-mediated hydroxylation modification of asparaginyl residues on HIF-α, preventing it from binding to the co-activating protein p300/CBP [12, 20]. Hypoxia can inhibit both hydroxylation modes of HIF-1α, leading to HIF-1α accumulation. Generation of ROS under hypoxic conditions can inhibit PHD via cysteine (Cys) oxidation and promote stabilization of HIF-1α levels [21]. Accumulated HIF-1α translocates to the nucleus, heterodimerizes with HIF-1β and binds to the HRE in the promoter region of HIF target genes [12, 22]. Figure 1 illustrates the transcriptional regulation of HIF-1α under hypoxia and normoxia, respectively.

### Non-oxygen-dependent regulatory pathway of HIF-1α

HIF-1α is involved in several non-oxygen-dependent regulatory pathways, including the nuclear factor-κB (NF-κB) signaling pathway [23]. This pathway controls hypoxia-responsive inflammatory gene expression and can directly trigger HIF-1α transcription [24]. Inhibiting NF-κB using pyrrolidine dithiocarbamate, a selective inhibitor, halts the production of HIF-1 protein. Furthermore, HIF-1α exhibits negative feedback regulation of NF-κB, as seen in the inhibition of NF-κB-dependent genes in a mouse model of periapical lesions [25]. M2-type pyruvate kinase isozyme type M2 (PKM2) relocates to the nucleus and interacts with NF-κB, aiding the transcriptional activation of hypoxia-inducible factors [26].

Moreover, the mitogen-activated protein kinase (MAPK) pathway plays a significant role in regulating HIF-1α. MAPK can phosphorylate HIF-1α, facilitating the binding and degradation of phosphorylated HIF-1α to pVHL, leading to increased HIF-1α accumulation [27]. Additionally, MAPK signaling influences HIF activity by promoting the formation of the HIF-p300/CBP complex and regulating the trans-activating activity of p300/CBP[28].

The phosphatidylinositol 3-kinase/protein kinase B (PI3K/Akt) signaling pathway is activated by hypoxia in a cell type-specific manner [29]. This pathway regulates protein synthesis and drives HIF-1α synthesis through components such as mammalian Target of Rapamycin (mTOR) and signal transducer and activator of transcription 3 (STAT3) [30].

Additionally, the Mousedouble minute 2 (Mdm2) pathway regulates HIF-1α. Under normoxic conditions, HIF-1α binds to p53 and promotes Mdm2-mediated ubiquitination and subsequent proteasomal degradation of HIF-1α [13, 31]. This pathway contributes to elevated levels of HIF-1α protein in hypoxic cells, thereby positively regulating the transcriptional activation of HIF-1 target genes and vascular endothelial growth factor (VEGF) in tumor cells during hypoxia [32]. Upon the accumulation of HIF-1α, it forms a heterodimer with HIF-1β, binding to the HRE in the promoter region of target genes, thereby activating downstream gene transcription.

## Hypoxia-induced modulation of HIF-1α in sepsis

### Respiratory failure and HIF-1α

ARDS is a complex syndrome characterized by heightened permeability of pulmonary capillary endothelial and alveolar epithelial cells, often leading to severe respiratory failure [33]. Bioinformatics research has pinpointed HIF-1α messenger ribonucleic acid (mRNA) as a potential autophagy-related gene associated with sepsis-associated ARDS [34]. Within endothelial cells, HIF-1α plays a pivotal role in supporting vascular repair and regression of inflammation in ARDS through the Forkhead Box Protein M1 (FOXM1) signaling pathway [35, 36]. Mice exhibiting suppressed HIF-1α demonstrate compromised vascular repair, persistent inflammatory response, and elevated mortality rates [35]. Furthermore, in a mouse model of sepsis-induced lung injury, the PHD inhibitor roxadustat showed promise in alleviating sepsis-induced acute lung injury [37].

### Circulatory failure and HIF-1α

In sepsis, patients often encounter both macrocirculatory and microcirculatory failures, resulting in impaired local tissue oxygenation. On the macro-level, HIF-1α plays a pivotal role in enhancing the myocardial tissue's tolerance to ischemic injury [8]. Diabetic mice deficient in HIF-1α exhibit significant cardiac contractile dysfunction, increased cardiac sympathetic innervation, and subsequent myocardial structural remodeling [38]. The expression of HIF-1α in myeloid cells provides protection during ischemia and reperfusion injury in the heart [39]. Moreover, HIF-1α alleviates myocardial inflammatory injury induced by coronary microembolization and enhances cardiac function by inhibiting the activation of the TLR4/MyD88/NF-κΒ signaling pathway [40]. Upregulation of Inducible Nitric Oxide Synthase (iNOS) may have a significant role [41]. HIF-1α can upregulate iNOS levels, thereby attenuating myocardial ischemia–reperfusion injury [8]. Additionally, treating rats with myocardial ischemia using recombinant adeno-associated virus expressing HIF-1α improves cardiac function and enhances cardiac capillary density [42]. While initially, high expression of HIF-1α protects cardiac function, prolonged elevation may negatively impact the heart. Long-term elevation of HIF-1α levels results in lipid accumulation, myocardial fibrosis, remodeling, and ultimately heart failure in mouse cardiomyocytes [43]. HIF-1α also influences the microcirculatory system. HIF-1α signaling increases nitric oxide (NO) production through the modulation of iNOS, whereas HIF-2α inhibits NO production [44]. Since NO acts as a vasodilator and vascular tone modulator, it can lead to a decrease in blood pressure. Additionally, elevated expression levels of both HIF-1α and HIF-2α are linked to increased microthrombosis in the lungs of mice [45].

### Cytopathic hypoxia

Studies have shown that pro-inflammatory cytokines can activate the oxygen-linked pathway through ROS-related mechanisms [46, 47]. In a mouse model of endotoxemia, lipopolysaccharide (LPS) exacerbated mechanical ventilation-induced diaphragmatic dysfunction and mitochondrial damage, partially through the HIF-1α signaling pathway [48]. Mitochondrial dysfunction was observed to reduce HIF-1α protein synthesis in HepG2 cells [49]. This finding may elucidate the escalation of mitochondrial dysfunction and reduction in HIF-1α levels during the middle and late stages of sepsis.

### Sepsis-induced anemia can result in tissue hypoxia

Sepsis-related anemia often results from factors such as iatrogenic blood loss, reduced plasma iron levels, diminished EPO production, shortened erythrocyte lifespan, and malnutrition [50]. In sepsis-associated anemia, the reduction in hemoglobin volume and oxygen-carrying capacity leads to lowered oxygen levels and inadequate oxygen delivery to tissues and cells. Furthermore, impaired erythrocyte deformability in sepsis patients can exacerbate microcirculatory blood flow disturbances [50]. The presence of anemia can trigger HIF-1α signaling through hypoxic and neuronal nitric oxide synthase (nNOS)-dependent mechanisms [51]. Given that HIF-1α stimulates EPO production, considering HIF-1α signaling as a potential therapeutic target in renal anemia is plausible. Clinical trials have explored the application of various hypoxia-inducible factor stabilizers for treating anemia in chronic kidney disease [52].

## Infection-induced modulation of HIF-1α in sepsis

### The role of HIF-1α in the context of bacterial sepsis

**Fig. 2:**
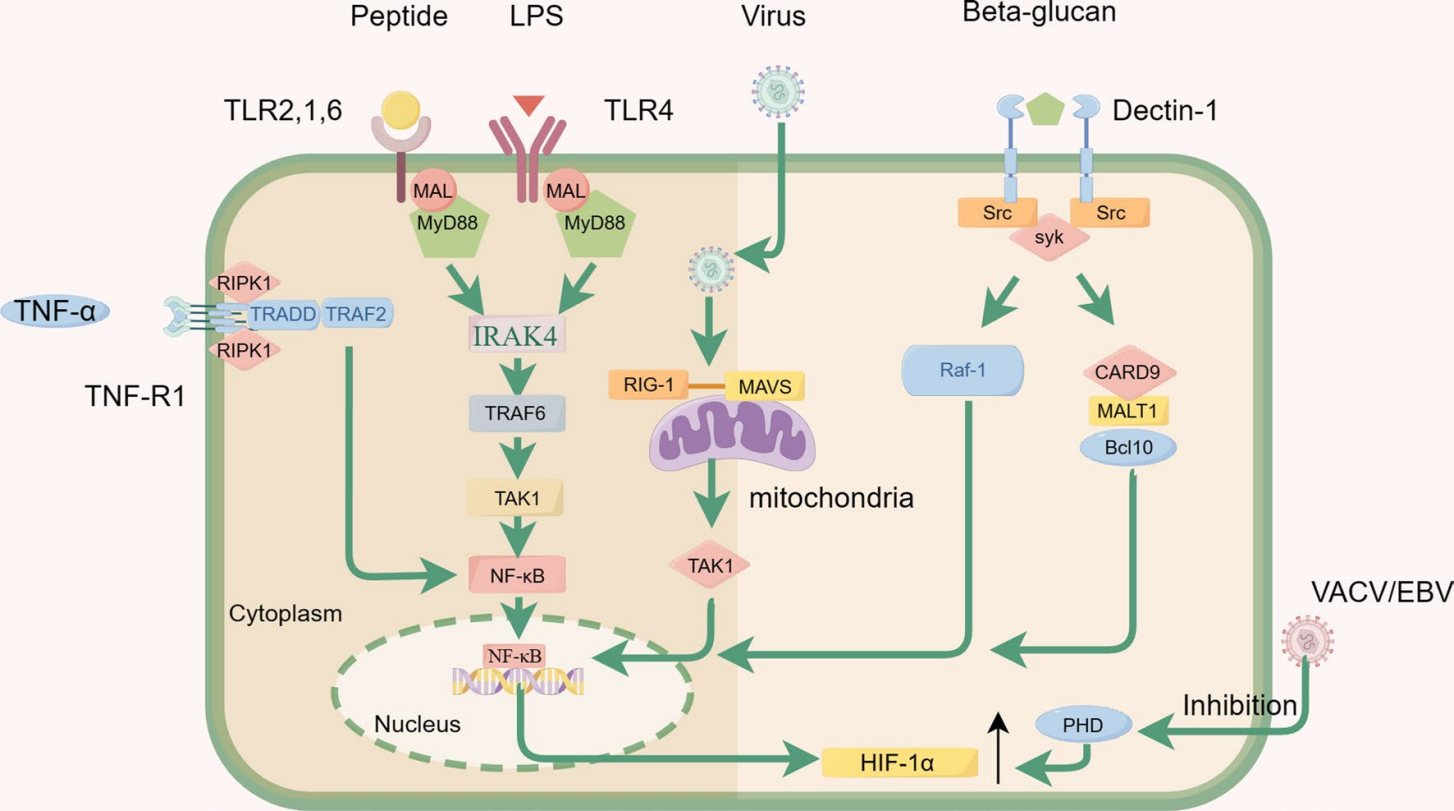
The infection of endothelial cells by pathogenic microorganisms triggers the activation of the NF-κB pathway, resulting in increased levels of HIF-1α. The figure mainly illustrates the pathways through which bacteria, viruses, fungi, and cytokines activate the NF-κB pathway: (1) The TLR activation by bacterial infection recruits the adaptor protein MyD88 (myeloid differentiation primary response 88) to propagate downstream signals. MyD88 subsequently activates a series of kinases, leading to the activation and nuclear translocation of nuclear factor-κB (NF-κB), a transcription factor that regulates the expression of several pro-inflammatory genes. (2) RIG-I plays a crucial role in initiating the innate antiviral immune response by serving as a key pattern recognition receptor for host recognition of viruses. RIG-I recognizes the RNA component of viruses and transmits signals by interacting with the downstream signaling molecule MAVS through its own CARD. This process activates the cellular transcription factors IRF-3 and NF-κB, allowing them to enter the nucleus. (3) Recognition of β-1–3-glucan in the fungal cell wall by dectin-1 enables the sensing of fungal pathogens and initiates host immune responses. Dectin-1 triggers the downstream signaling pathways of Syk and Raf1, which subsequently modulate the activation of both classical and non-classical NF-κB signaling pathways. (4) Upon activation by TNF-α, the TNFR1 triggers the formation of a signaling complex that induces a cellular response. In the assembly of Complex I, the activated TNFR1 binds to TRADD (TNFRSF1A-associated via death domain), followed by the interaction with a variety of components, such as receptor-interacting protein kinase 1 (RIPK1). This signaling pathway activates NF-κB and MAPK

Elevated HIF-1α levels manifest in bacterial sepsis, with the immune response to diverse bacterial pathogens such as *Streptococcus pyogenes*, *Staphylococcus aureus*, and *Pseudomonas aeruginosa* serves as a stimulant for the augmentation of HIF-1α levels [9, 53]. Various potential mechanisms underlie the elevation of HIF-1α in response to infection. Firstly, tissue inflammation induces local tissue hypoxia, attributed to heightened cellular oxygen consumption resulting from bacterial infection, as well as the migration and proliferation of immune cells at the infection site—a phenomenon known as inflammatory hypoxia [54, 55]. Secondly, distinct bacterial components, such as outer membrane proteins, Baltonsomal Adhesin A, or LPS from *Escherichia coli*, have been found to contribute to the upregulation of HIF-1α levels [56]. Particularly, LPS from *Escherichia coli* has been associated with the stability of HIF-1α, increasing its levels in macrophages through the activity of mitogen-activated protein kinase (MAPK) and NF-κB signaling pathways [57–59]. Moreover, cytokines released by immune cells post-infection, including IL (interleukin) -6, -4, -12, -1, and tumor necrosis factor alpha (TNF-α), can contribute to the increased expression of HIF-1α. Table 1 demonstrates the role of HIF-1α in major pathogenic microbial infections. Figure 2 illustrates the process of infection of endothelial cells by different pathogenic microorganisms.

**Table 1:** Role of HIF-1α in different pathogenic microbial infections

Pathogenic microorganism	Model	The role of HIF-1α in infections	References
*Bacteria*			
*Escherichia coli*	UTI model	Promotes the production of NO and antimicrobial peptides	[71]
*Pseudomonas aeruginosa*	Keratitis model	Enhances the activation of inflammatory cells, production of antimicrobial peptides, and ability to kill bacteria	[72]
*Klebsiella pneumoniae*	Pneumonia model	HIF-1α is a susceptibility factor for bacterial invasion during pneumonia	[73]
*Clostridium difficile*	Ileal loop model	Protects the intestinal mucosa from C difficile toxins	[74]
*Staphylococcus aureus*	Kidney abscesses model	Participation in abscess formation	[75]
*Streptococcus pneumoniae*	Pneumonia model	No significant impact	[76]
*Salmonella Typhimurium*	Salmonella infection model	No significant impact	[77]
*Viruses*			
BKV	Kidney tissue samples	bind the BKV promoter and enhance BKV replication	[78]
RSV	Primary human small alveolar epithelial cells	RSV replication and the glycolytic pathway	[79]
DENV	Primary monocytes	Enhance antibody‐dependent DENV infection in monocytic cells	[80]
HBV	Liver-derived cell	Increases HBV RNA transcript levels, core protein levels, cytoplasmic localization of core protein, and replication of the HBV	[81]
VACV	HEK293T cell	Involved in virus-induced hypoxic responses	[61]
SARS-CoV-2	PBMCs	Virus replication and monocyte cytokine production	[82]
*Fungi*			
*Aspergillus fumigatus*	A549 cells and mouse airway cells	Upregulation induces pro-inflammatory factors	[70]
*Candida albicans*	CA-colonized mice	Inhibits *Candida albicans* colonization	[69]

*BKV* BK polyomavirus, *CA* Candida albicans, *DENV* Dengue virus, *HBV* hepatitis B virus, *NO* nitric oxide, *PBMCs* Peripheral blood mononuclear cells, *RSV* respiratory syncytial virus, *UTI* urinary tract infections, *VACV* Vaccinia Virus

### The role of HIF-1α in the context of viral sepsis

In the context of viral sepsis, elevated levels of HIF-1α are evident in response to viral infections. Several factors contribute to the increased levels of HIF-1α. Firstly, specific viruses can accumulate HIF-1α during infection through inducing the degradation of prolyl hydroxylase (PHD) [60]. For example, the vaccinia virus (VACV) can inhibit the PHD2-dependent hydroxylation pathway of HIF-1α by binding to PHD2 via the C16 protein post-organismal infection, expediting the rapid accumulation of HIF-1α [61]. A similar mechanism has been observed in the Epstein–Barr virus (EBV), where the Latent Membrane Protein 1 (LMP1) of EBV induces the degradation of PHD1 and PHD3, leading to an upregulation of HIF-1α [62]. Secondly, activated inflammatory pathways during the inflammatory response can induce increased levels of HIF-1α. Pattern recognition receptors (PRRs) activate nonspecific innate immunity by recognizing specific endogenous or exogenous ligands, recruiting inflammatory cell aggregates, activating inflammatory factor pathways, and releasing inflammatory factors [63]. Finally, some respiratory viral infections can directly damage lung tissue, leading to hypoxia.

### The role of HIF-1α in the context of fungal sepsis

Hypoxia assumes a pivotal role in shaping the host microenvironment during fungal infections [64]. Research has documented hypoxic conditions in infected tissues in mouse models of both *Candida albicans* and *Aspergillus* infections [65, 66]. The emergence of infection foci or biofilms by host cells and fungi at the site of infection leads to the emergence of hypoxia [67]. This local hypoxia is exacerbated by structural damage to the vascular system, resulting in reduced oxygen delivery. The restricted availability of oxygen induces a hypoxic response in the fungi, ultimately contributing to increased levels of HIF-1α [68]. HIF-1α plays a protective role in fungal infections by effectively reducing Candida albicans colonization in the gastrointestinal tract, as demonstrated in research [69]. Furthermore, the upregulation of HIF-1α has been shown to alleviate airway inflammation in a mouse model exposed to Aspergillus fumigatus [70].

## Immune-induced modulation of HIF-1α in sepsis

### Inflammatory mediator-induced modulation of HIF-1α in sepsis

Immunologically, sepsis is initiated by the simultaneous recognition of various infection-derived microbial products and endogenous danger signals by the complement system and specific cell-surface receptors, closely linked to inflammatory dysregulation [63]. Pathogen-associated molecular patterns (PAMPs) released by invading pathogenic microorganisms trigger the immune response and prompt immune cells to release a spectrum of inflammatory factors, leading to a cytokine storm and activation of the innate immune system [1]. Furthermore, injuries such as sepsis, trauma, and burns result in the release of endogenous pattern recognition receptor agonists, known as damage-associated molecular patterns (DAMPs), which in turn induce an inflammatory response [83]. These interlocking positive feedback loops between PAMPs, DAMPs, and their receptors can serve as the molecular basis for the systemic inflammatory response initiated by infection, as well as damaged tissues or non-specific stressors [41]. Indeed, multiple inflammatory factors could modulate the levels of HIF-1α. This modulation of HIF-1α levels by inflammatory factors plays a significant role in various physiological and pathological processes. Table 2 details the effects of common inflammatory factors on HIF-1α levels.

**Table 2:** Major inflammatory factors that modulate HIF-1α

Inflammatory factors	Model	Molecular mechanism/signaling pathway	Regulation of HIF-1α	References
IL-1β	In vitro	NF-κΒ/COX-2	Up	[90]
IL-2	In vitro	PI3K/AKT	Up	[91]
IL-4	In vitro	PI3K/AKT	Up	[92]
IL-6	In vitro	STAT3	Up	[93]
IL-8	Mouse fatty liver model	PI3K/AKT	Up	[94]
IL-10	Rat hypoxia model	Not given	None	[95]
IL-13	In vitro	Induce hypoxia signaling pathway genes	Up	[96]
IL-15	In vitro	STAT3	Up	[97]
IL-17	MTB Mouse model	Not given	Down	[98]
IL-18	Bioinformatics analysis	NF-κB	Up	[99]
IL-27	In vitro	STAT1	Down	[100]
IL-33	Murine model of HPH	IL-33/ST2/HIF-1α	Up	[101]
IL-34	Clinical research	Not given	Up	[102]
IL-37	PDAC	Inhibition of STAT3	Down	[103]
IL-38	CIA rats	SIRT1/HIF-1α	Down	[104]
TNF-α	In vitro	IKKβ	Up	[105]
IFN	In vitro	PI3K/AKT	Up	[106]
NO	In vitro	PI3K/AKT	Up	[107]
ROS	In vitro	Inactivate PHD	Up	[108]

*CIA* collagen-induced arthritis, *HPH* hypoxia-induced pulmonary hypertension, *IKKβ* I kappa B kinase beta, *MTB* Mycobacterium tuberculosis, *PDAC* pancreatic ductal adenocarcinoma, *Ref* reference, *ROS* Reactive Oxygen Species

### Immune cell-induced modulation of HIF-1α in sepsis

HIF-1α plays a pivotal role in regulating the innate immune system. The suppression of the HIF gene in myeloid cells reduces ATP production, leading to a notable decrease in inflammatory responses [9]. Consequently, macrophages exhibit reduced invasiveness and motility, coupled with impaired bacterial clearance within macrophages [9]. Additionally, HIF-1α enhances cellular antimicrobial activity by promoting the formation of extracellular traps in mast cells [84]. Moreover, HIF-1α modulates the survival, function, and activity of dendritic cells. Under hypoxic conditions, increased HIF-1α levels in immature dendritic cells promote apoptosis, while in mature dendritic cells, it alleviates hypoxia-induced cell death [85].

A complex interplay exists between HIF-1α and the adaptive immune system, with both activating and inhibitory effects on immune cell regulation influenced by the cellular milieu and specific conditions [86]. Notably, HIF-1α exerts an inhibitory influence on T lymphocytes, as demonstrated by research indicating that enhanced activation of the HIF pathway effectively suppresses T cell proliferation in myeloid/T cell co-cultures [87]. This suppression may contribute to the reduced proliferation of lymphocytes observed with increased HIF-1α levels in early sepsis. Studies have revealed an accumulation of B lymphocytes in mice with specific deficiencies in HIF-1α expression [88]. However, HIF-1α also plays a critical role in immune cell activation. Genetic abnormalities in the HIF-1α gene lead to disruptions in glycolysis and energy metabolism in B cells, resulting in altered cell differentiation and autoimmunity [89].

### Regulation of HIF-1α by iron metabolism in sepsis

The hydroxylation of HIF-1α is facilitated by prolyl hydroxylase (PHD) in the presence of oxygen, divalent iron, 2-oxoglutarate (2-OG), and ascorbic acid, targeting the proline residues of HIF-1α [23]. In septic patients, pathogenic microorganisms competitively acquire iron within the host by processes such as elemental metal import, removal of metal by iron carriers from extracellular sites, and acquisition from host proteins [108]. The sequestration of iron by pathogenic microorganisms impacts the PHD-mediated hydroxylation of HIF-1α, leading to the accumulation of HIF-1α. This process may contribute to the elevated levels of HIF-1α observed in sepsis.

## Potential targeted drugs

Given its involvement in critical aspects of sepsis, HIF-1α has emerged as a potential therapeutic target for treating sepsis in humans. The pharmacological effects of HIF-1α include the stimulation of erythropoiesis, modulating inflammatory factors, reprogramming cell metabolism during hypoxia, and influencing the body's adaptive response to ischemia and inflammation [19].

### Targeted therapies for HIF-1α upregulation

The medications that stimulate HIF-1α upregulation can be broadly classified into direct HIF-1α inducers and PHD inhibitors [36, 37, 74, 109–113]. Acetate, a significant short-chain fatty acid produced by gut microbes, enhances HIF-1α levels by triggering increased glycolysis, thereby improving macrophage killing through the HIF-1α/IL-1β axis [109]. Moreover, the HIF-1α agonist mimosine enhances phagocytosis, increases bactericidal capacity, and reduces lesion severity in a murine model of Staphylococcus aureus skin infection [111]. Established pharmaceuticals, known for their clinical efficacy, also exhibit modulation of HIF-1α. For instance, rabeprazole acts as a potent HIF-1α inducer, facilitating vascular repair and reducing sepsis-induced lung inflammation through the endothelial HIF-1α/FoxM1 signaling pathway [36].

Several PHD inhibitors have demonstrated symptomatic and prognostic benefits in animal models of sepsis or infection. Roxadustat (FG-4592), a transient small-molecule PHD inhibitor, increases HIF-1α expression in the lungs through the HIF-1α/heme oxygenase-1 (HO-1) signaling pathway, ameliorating LPS-induced lung injury and inflammation [37]. Dimethyloxaloylglycine (DMOG), another PHD inhibitor, creates a hypoxic microenvironment by inhibiting PHD enzyme activity, stabilizing HIF-1α, and impacting various intracellular signaling pathways. DMOG has been found to alleviate toxin-induced intestinal inflammation, maintain epithelial barrier function, and protect against C. difficile toxin-induced intestinal injury [74]. Additionally, AKB-4924, a PHD inhibitor with no direct antibacterial activity, elevates HIF-1α levels and inhibits the proliferation of S. aureus, reducing lesion formation in a murine skin abscess model [110]. The potential therapeutic application of AKB-4924 is promising due to the reduced risk of developing bacterial resistance. Table 3 details the primary medications and compounds that upregulated the expression of HIF-1α.

**Table 3:** Medications for treating sepsis and its complications by increasing HIF-1α expression

Drug Names	The role of HIF-1α	Modulation of HIF-1α	Model	References
Rabeprazole	Facilitates vascular repair and resolution of lung injury	Up (directly HIF-1α pathway)	LPS-induced sepsis mouse	[36]
Roxadustat	Mitigates sepsis-induced acute lung injury	Up (PHD inhibitor)	I/R-induced AKI mouse mice	[37]
Acetate	Improved killing of *S. pneumoniae* by alveolar macrophages	Up (directly HIF-1α pathway)	*S. pneumoniae* infection mouse model	[109]
DMOG	Reducing *Clostridium difficile* toxin-induced intestinal damage	Up (PHD inhibitor)	Mouse ileal loop model	[74]
AKB-4924	Enhances the cutaneous innate defenses against bacterial infections	Up (PHD inhibitor)	Mouse skin abscess model	[110]
Mimosine	Enhancement of the bactericidal capacity of phagocytes	Up (directly HIF-1α pathway)	*S. aureus* skin infection model	[111]
Edaravone	Exerts cardioprotective effects	Up (directlyHIF-1 α/HO-1 pathway)	CLP-induced sepsis rats	[112]
Phlorizin	Improve sepsis-induced cardiomyocyte injury	Up (induces the oxygen deprivation)	SIMD mouse model	[113]

*AKI* acute kidney injury, *CLP* cecal ligation and puncture, *DMOG* Dimethyloxaloylglycine, *HCP* Houttuynia cordata polysaccharide, *I/R* ischemia/reperfusion, *Ref*. reference, *S. aureus Staphylococcus aureus*, *S. pneumoniae Streptococcus pneumoniae*, *SIMD* Sepsis-induced myocardial dysfunction

### Targeted therapies for HIF-1α downregulation

HIF-1α activity inhibitors can be categorized into distinct groups based on their mechanisms of action [114]: (1) those affecting the degradation of HIF-1α; (2) those inhibiting the DNA transcription and expression of HIF-1α; (3) those blocking mRNA translation; (4) those preventing the binding of HIF-1α and Hypoxia-Response Element (HRE); and (5) those disrupting the formation of HIF-1α transcriptional complexes, among others. In the context of sepsis, medications that reduce HIF-1α activity typically function by inhibiting the DNA transcription and expression of HIF-1α.

Various drugs demonstrate protective effects against sepsis-induced damage in different target organs by modulating HIF-1α activity [115–139]. For instance, lidocaine mitigates the inflammatory cascade induced by HIF-1α by inhibiting the NF-κB signaling pathway, effectively downregulating HIF-1α transcription and expression [115]. Houttuynia cordata polysaccharide (HCP) also displays inhibitory effects on HIF-1α DNA transcription and expression, offering beneficial effects in H1N1-induced intestinal injury by modulating the TLR4 pathway, reducing HIF-1α expression, and preserving tight junction proteins such as zonula occludens-1 (ZO-1) [116]. Cynaroside suppresses hepatic inflammation by inhibiting PKM2/HIF-1α interactions, resulting in decreased activation of HIF-1α target genes and facilitating the transition of M1 macrophages to M2 macrophages [118]. Norisoboldine hinders the translocation of PKM2 from the cytoplasm to the nucleus, reducing HIF-1α expression and mitigating sepsis-induced acute lung injury [119]. Additionally, resveratrol enhances endothelial nitric oxide synthase (eNOS) expression and lowers HIF-1α levels to enhance vasodilatory function in a septic shock model [125]. Table 4 outlines the primary medications and compounds that downregulate the expression of HIF-1α.

**Table 4:** Drugs targeting sepsis and complications by down-regulation of HIF-1α

Drug Names	The role of HIF-1α	Modulation of HIF-1α	Model	References
Lidocaine	Inhibiting glycolysis to attenuate inflammatory response	Down (indirectly through NF-κB pathway)	LPS-induced sepsis mouse	[115]
HCP	Reduce intestinal damage in H1N1 virus-infected individuals	Down (indirectly through TLR4 pathway)	H1N1 virus infected mouse mice	[116]
XJDHT	Participation in aerobic glycolysis in sepsis	Down (indirectly through TLR4 pathway)	CLP-induced sepsis rats	[117]
Cynaroside	Attenuates liver injury	Down (indirectly through PKM2 pathway)	CLP-induced sepsis mouse	[118]
Norisoboldine	Mitigates sepsis-induced acute lung injury	Down (indirectly through PKM2 pathway)	LPS-induced sepsis mouse	[119]
LBP	Altering glycolysis and the M1 differentiation of macrophages	Down (indirectly through PKM2 pathway)	LPS-induced macrophages model	[120]
Cya	Affects B cell Migration	Down (stimulate PHD activity)	Human and mouse B cell	[121]
AV	Promoting inflammatory responses	Down (stimulate PHD2 activity)	CLP-induced sepsis mouse	[122]
TIIA	Promoting inflammatory responses	Down (indirectly through PI3K and MAPK pathway)	LPS-induced lung injury mouse	[123]
Propofol	Promoting inflammatory responses	Down (indirectly through MAPK pathway)	Mouse model of endotoxemia	[124]
Resveratrol	Improvement of vasodilatory function in a septic shock model	Down (not given)	CLP-induced septic shock rats	[125]
Chicoric acid	Mediated glycolysis and mitochondrial oxidative burst	Down (reducing ROS production)	LPS-induced sepsis mouse	[126]
TASE	Leading to endotoxin tolerance in sepsis monocytes	Down (indirectly through IRAK-M pathway)	Monocytes in patients with sepsis	[127]
AmB	Regulation of EPO expression	Down (reinforcing FIH-mediated repression)	Hypoxia and anemia rats	[128]
Rosmarinic acid	Regulation of LPS-induced microglial M1 polarization	Down (indirectly through RACK1 pathway)	CLP-induced sepsis mouse	[129]
2ME2	Promoting inflammatory responses	Down (reduced HIF-1α activity)	CLP- and LPS-induced sepsis mouse	[130]
Emodin	Modulates intestinal barrier injury	Down (inhibits the expression of HIF-1α)	LPS-induced intestinal epithelial cells model	[131]
N5P	Promoting inflammatory responses	Down (inhibits the expression of HIF-1α)	ALI rat model	[132]
Valproic acid	Participation in burn-induced gut barrier dysfunction	Down (inhibits HIF-1α accumulation)	Rat burn model	[133]
DHMF	Promoting inflammatory responses	Down (inhibits HIF-1α accumulation)	LPS-induced lung injury mouse	[134]
Eriocitrin	Regulation of glycolysis in sepsis	Down (inhibition of *HIF-1α* mRNA)	LPS-induced sepsis-associated ALI mouse	[135]
Metformin	Promoting inflammatory responses	Down (inhibition of *HIF-1α* mRNA)	LPS-induced sepsis-associated liver injury mouse	[136]
Landiolol	Involved in sepsis-related AKI	Down (inhibition of *HIF-1α* mRNA)	Mouse model of endotoxaemia	[137]
Enoxaparin	Causes diaphragm damage	Down (inhibition of HIF-1α mRNA and Protein)	LPS-induced sepsis mouse	[138]
Dexmedetomidine	Participation in aerobic glycolysis in sepsis	Down (inhibition of HIF-1α mRNA and Protein)	LPS-treated macrophages	[139]

*AKI* acute kidney injury, *ALI* acute liver injury, *AmB* Amphotericin B, *AV* Adhatoda Vasica, *CLP* Cecal ligation and puncture, *Cya* Cyclosporine A, *DHMF* 5,7-dihydroxy-8-methoxyflavone, *LBP* Lycium barbarum polysaccharide, *LPS* Lipopolysaccharide, *N5P* N-phenethyl-5-phenylpicolinamide, *Ref.* reference, *ROS* reactive oxygen species, *TASE* Thiosulfinate-enriched Allium sativum extract, *TIIA* tanshinone IIA, *XJDHT* Xijiao Dihuang decoction, *2ME2* 2-Methoxyestradiol

### Clinical value of HIF-1α

Recent clinical studies have shifted focus toward translating HIF-1α research findings from basic science to clinical applications, emphasizing its relevance in post-diagnostic and prognostic aspects of sepsis. In a prospective study comparing HIF-1α Mrna levels in the blood of healthy volunteers and patients in shock, significantly elevated levels of HIF-1α Mrna were observed in shock patients compared to healthy volunteers [140]. Similarly, serum HIF-1α levels in intensive care patients exhibited diagnostic potential in sepsis, with significantly higher concentrations detected in patients with septic shock, septic non-shock, and infection groups than in those undergoing elective surgery (160.39 ± 19.68 vs 135.24 ± 20.34 vs 114.34 ± 15.50 vs 113.37 ± 15.50 pg/Ml, respectively, *P* < 0.01) [141]. Further research highlighted the use of HIF-1α, combined with other clinical parameters, as a tool for sepsis diagnosis, demonstrating high diagnostic accuracy (AUC 0.926, 95% CI 0.885–0.968) and revealing a U-shaped relationship between HIF-1α levels and ICU mortality [15].

In contrast, some studies have presented conflicting results. A prospective study reported a significant decrease in HIF-1α expression levels in septic patients compared to healthy volunteers [140]. This discrepancy may be attributed to LPS tolerance, where repetitive inflammatory or hypoxic stimuli initially upregulate the expression of inflammatory genes, followed by subsequent suppression of their expression levels [15]. Additionally, experiments with LPS-stimulated neutrophils indicated an initial rise in HIF-1α protein levels after 4 h of LPS stimulation, followed by a gradual and significant decline [142].

## Conclusion and prospect

HIF-1α plays a crucial role in sepsis, and its activation is closely tied not only to intracellular hypoxia but also to the inflammatory process and immune regulation. During sepsis, the activation of HIF-1α governs the host’s adaptive response to hypoxia and influences the release of inflammatory mediators, as well as the balance between anti-inflammatory and immune tolerance states. Furthermore, HIF-1α activation is implicated in regulating a spectrum of pathophysiological processes, including mitochondrial function and apoptosis. Future studies can explore the molecular mechanisms and pathways of HIF-1α in sepsis, with the potential to reveal new targets and strategies for the early diagnosis and treatment of sepsis.

## Data Availability

Not applicable.

## Abbreviations

2-OG: 2-Oxoglutarate
2ME2: 2-Methoxyestradiol
α-KG: Alpha-ketoglutarate
AKI: Acute kidney injury
ALI: Acute liver injury
AmB: Amphotericin B
ATP: Adenosine triphosphate
AV: Adhatoda vasica
Bhlh-PAS: Basic Helix-Loop-Helix-Periodicity-Aryl Hydrocarbon Receptor Nuclear Translocator-Single-Minded
BKV: BK polyomavirus
CA: Candida albicans
CBP: Cyclic adenosine monophosphate-response binding protein binding protein
CIA: Collagen-induced arthritis
CLP: Cecal ligation and puncture
Cys: Cysteine
Cya: Cyclosporine A
DAMPs: Damage-associated molecular patterns
DNA: Deoxyribonucleic acid
DENV: Dengue virus
DHMF: 5,7-Dihydroxy-8-methoxyflavone
EBV: Epstein–Barr virus
Enos: Endothelial nitric oxide synthase
EPO: Erythropoietin
FOXM1: Forkhead Box Protein M1
FIH: Factor-inhibiting HIF-1
HAT: Histone acetyltransferase
HIF: Hypoxia-inducible factor
HIF-1α: Hypoxia-inducible factor-1α
HPH: Hypoxia-induced pulmonary hypertension
IKKβ: I kappa B kinase beta
IAV: Influenza A virus
I/R: Ischemia/reperfusion
Inos: Inducible nitric oxide synthase
JAKs: Janus Kinases
LBP: Lycium barbarum polysaccharide
LPS: Lipopolysaccharide
mTOR: Mammalian Target of Rapamycin
MAPK: Mitogen-activated protein kinase
Mdm2: Mousedouble minute 2
mRNA: Messenger ribonucleic acid
MTB: Mycobacterium tuberculosis
N5P: N-phenethyl-5-phenylpicolinamide
NF-κB: Nuclear factor-κB
NH2-terminal domains: N-TAD
NK: Natural killer
NO: Nitric oxide
ODDD: Oxygen-dependent degradation domain
PDAC: Pancreatic ductal adenocarcinoma
PAMPs: Pathogen-associated molecular patterns
PBMCs: Peripheral blood mononuclear cells
PHD: Prolyl hydroxylase
PHD: Prolyl hydroxylases domain
PI3K/Akt: Phosphatidylinositol 3-kinase/protein kinase B
PRR: Pattern recognition receptors
PKM2: Pyruvate kinase isozyme type M2
PSM: Propensity score matching
ROS: Reactive oxygen species
RSV: Respiratory syncytial virus
Ref: Reference
SIMD: Sepsis-induced myocardial dysfunction
S. aureus: Staphylococcus aureus
S. pneumoniae: Streptococcus pneumoniae
STAT3: Signal transducer and activator of transcription 3
STATs: Signal transducers and activators of transcription
TAD: Transactivation domains
TASE: Thiosulfinate-enriched *Allium sativum* extract
TIIA: Tanshinone IIA
UTI: Urinary tract infection
VACV: Vaccinia virus
VEGF: Vascular endothelial growth factor
XJDHT: Xijiao Dihuang decoction
ZO-1: Zonula occludens-1
